# ALDH, SOX and CD147 Εxpression in Oral Lichen Planus Compared to Oral Squamous Cell Carcinoma Epithelium: An Immunohistochemical Study

**DOI:** 10.7759/cureus.114268

**Published:** 2026-08-10

**Authors:** Vasileios Zisis, Petros Papadopoulos, Christina Charisi, Konstantinos Poulopoulos, Ioannis Fotopoulos, Theodoros Lillis, Nikolaos Dabarakis, Georgios Parlitsis, Nikolaos Shinas, Pavlos Theodosiadis, Konstantinos Paraskevopoulos, Konstantinos Vahtsevanos, Athanasios Poulopoulos

**Affiliations:** 1 Oral Medicine and Pathology, European University Cyprus, Nicosia, CYP; 2 Oral Medicine and Pathology, Aristotle University of Thessaloniki, Thessaloniki, GRC; 3 Paediatric Dentistry, Aristotle University of Thessaloniki, Thessaloniki, GRC; 4 Dentoalveolar Surgery, Implantology and Oral Radiology, School of Dentistry, Faculty of Health Sciences, Aristotle University of Thessaloniki, Thessaloniki, GRC; 5 Dentoalveolar Surgery, Implantology and Oral Radiology, School of Dentistry, Faculty of Health Sciences, Aristotle University of Thessaloniki, Thessaloniki, GRC, Thessaloniki, GRC; 6 Mechanical Engineering, Eindhoven University of Technology, Eindhoven, NLD; 7 Oral and Maxillofacial Radiology, Henry M. Goldman School of Dental Medicine, Boston University, Boston, USA; 8 Oral and Maxillofacial Surgery, Papanikolaou Hospital, Aristotle University of Thessaloniki, Thessaloniki, GRC

**Keywords:** aldh, biomarkers, cancer stem cells, cd147, emmprin, oral lichen planus, oral squamous cell carcinoma, sox

## Abstract

Introduction: Oral potential malignant diseases (OPMDs) are transformed into oral squamous cell carcinoma (OSCC) by cancer stem cells (CSCs), which also start the process of carcinogenesis de novo. Our study's objective was to examine the expression of ALDH, SOX, and CD147 in OSCCs and oral lichen planus (OLP).

Methods: An immunohistochemical detection was performed in 24 OLP samples (10 reticular OLPs (ROLPs) and 14 erosive OLPs (EOLPs)) and 21 OSCC samples of all differentiation levels to track the expression profile of ALDH1/2, SOX2, and CD147. Immunohistochemistry was applied in a semiquantative fashion to finish the aforementioned process. The paraffin-embedded tissue samples were taken from biopsies conducted in the Department of Oral Medicine/Pathology, School of Dentistry, Aristotle University of Thessaloniki, Greece, between 2014 and 2019, as well as from the Oral and Maxillofacial Surgery Clinic of G. Papanikolaou General Hospital, Aristotle University, and the Oral and Maxillofacial Surgery Clinic of St Luke Hospital, Thessaloniki, Greece. A scale ranging from 0 to 2 was used to assess the expressions of ALDH1/2, SOX2, and CD147 based on the proportion of positive epithelial cells. Depending on the sample size, either Fischer's exact test or Pearson chi-square test was used for the statistical analysis, with a significance threshold of p≤0.05.

Results: ALDH1/2 expression was statistically considerably greater in OSCC compared to EOLP (p=0.019) and ROLP (p<0.001), according to the statistical analysis. The statistical analysis of SOX2 staining revealed statistically significant greater expression in OSCC compared to ROLP (p=0.012) and EOLP compared to ROLP (p=0.024). The statistical analysis revealed that EOLP had statistically substantially higher expression of CD147 staining than ROLP (p=0.019). There were no statistically significant findings from the remaining comparisons.

In conclusion, the distinctively increased expression of CD147 in EOLP as opposed to ROLP raises the possibility that erosive lichenoid lesions contain neoplastic cells with specific CSC traits. The distinctively increased expression of SOX2 in OSCC and EOLP as opposed to ROLP raises the possibility that erosive lichenoid lesions contain malignant cells with specific CSC traits. In any event, additional experimental testing in a greater number of samples is necessary for the clinical application of CSC biomarkers as prognostic variables.

## Introduction

Oral lichen planus (OLP) is characterized as an autoimmune, chronic inflammatory illness with an unclear origin that is caused by T-lymphocyte aggressiveness against the oral epithelium's basal layer. OLP primarily manifests as white reticular lesions, which may or may not be followed by erosive, atrophic, or erythematous lesions [1]. OLP is now regarded as an oral potential malignant disease (OPMD). Based on data, studies have shown that OLP is a common condition that affects 1.01% of people worldwide and 1.43% of people in Europe [2]. This frequency rises noticeably and steadily starting at age 40 [2]. Similarly, evidence has shown that the disease's malignancy rate is 1.14% of cases [3].

However, it has been reported that one of the factors that most influences the risk of malignancy published for this disease is the methodological quality of the studies on the subject [4], with the studies with the highest methodological quality presenting a risk of OLP malignancy of 2.28%. The probability of OLP progression to cancer has been consistently underestimated, according to published research [5,6]. To enhance the design and methodological quality of OLP malignancy investigations, a number of criteria have been proposed [4,7]. Although the molecular processes behind the malignant transformation of OLP are mainly unclear, they appear to be connected to the carcinogenic consequences associated with the persistent inflammatory aggressiveness produced in this lesion, as in other autoimmune illnesses [6,8]. Generally speaking, the malignant transformation of OLP is most likely caused by the molecular changes that the inflammatory infiltrate causes in the basal cell layer of the attacked oral epithelium as well as the effects that the inflammatory infiltrate's chemokines and growth factors have on the basal cells, which then react by increasing their proliferative activity and inhibiting their apoptotic mechanisms [1]. All of this creates an environment that is conducive to the emergence of mutations that increase the risk of cancer. The risk of a final molecular event culminating in the appearance of a malignant clone extends to the entire oral mucosal surface affected by OLP, according to another pertinent conclusion. This would explain the high percentage of malignant OLP cases developing multiple oral carcinomas [9] and provide a molecular justification for the current consideration of OLP as a malignant field [10,11].

A tiny population of cancer stem cells (CSCs) with the capacity for self-renewal and pluripotent differentiation into several cancer cell types is thought to be responsible for the unchecked growth of tumor cells [12]. By generating new tumor cells, CSCs are thought to endure in malignancies and contribute to metastasis, treatment resistance, and post-operative recurrence. CSCs are resistant to numerous widely used therapies such as chemotherapy [13]. Therefore, focusing on CSCs ought to offer novel treatments to increase cancer patients' chances of survival and may reveal the genetic, epigenetic, and microenvironmental diversity of tumor cell populations. Most crucially, CSCs’ biomarkers may be applied to potentially malignant lesions, thus distinguishing the most perilous ones. The objective of our study is to identify such biomarkers, for possible clinical applications.

The presence of CSCs was initially discovered in acute myeloid leukemia in 1997 [14]. Glioblastoma [15], breast carcinomas [16,17], gastric cancer [18], and colorectal cancer [19] were subsequently discovered to include CSCs. Nevertheless, it is yet unknown what processes lead CSC intracellular dysregulation to malignancy. Therefore, biomarkers of these CSCs are essential for studying the ways in which CSCs might interact with the surrounding cells to develop into neoplasia. Furthermore, these markers are helpful in defining the cell fates of CSCs as well as their heterogeneity.

A metanalysis by Tripathi et al. in 2024 investigated the correlation between CSC marker expression and tumor characteristics, as well as their role in predicting the survival of subjects with oral carcinoma [20]. This metanalysis of 19 retrospective studies evaluated in particular the prognostic significance of CD44, CD24, CD133, and ALDH in oral squamous cell carcinoma (OSCC). The studies included a total of 1410 patients, with a mean sample size of 74.21. The results showed that CD133 expression was associated with poor prognosis, while ALDH expression was correlated with lymph node metastasis and clinical staging. In addition, CD24 expression was associated with a good prognosis, while CD44 expression was associated with lower overall survival rates [20]. However, the authors stated that the limitations of the study included the small sample size, which may have diminished the data's ability to identify any pattern as well as the events were calculated using data digitalization of Kaplan-Meier curves, which may have resulted in minor differences with the actual data due to manual errors [20].

In addition to the expected role of CSCs in malignant and potentially malignant oral epithelial lesions, a study by Balbinot et al. showed that they may play a role in odontogenic tumors as well [21]. The study verified the expression of SOX2, NANOG, and OCT4, stem cell biomarkers, in ameloblastoma (AME) parenchyma and subtle expression in the odontogenic epithelium of dentigerous cyst (DC) and dental follicle (DF), with high expression of these markers in AME neoplastic cells. The expression of SOX2, NANOG, and OCT4 was found to be increased in AME parenchyma compared to non-tumor epithelium. The authors suggested that these proteins may be involved in the progression and recurrence of AME. The expression of these markers is thought to be related to the tumor cell survival and the ability of the tumor to maintain its growth and expansion [21]. The limitations, however, of the study included the use of immunohistochemistry and immunofluorescence, which may not guarantee the full specificity of the antibody used. The study also had a limited sample size, which may affect the generalizability of the results.

Methylation may also interfere in normal processes. Han et al. investigated the epigenetic regulation of CSC surface markers, including CD24, CD44, CD133, and CD147, in OSCC and found that their transcriptional silencing is associated with promoter hypermethylation, which could be used as a biomarker for prognosis [22]. In particular, they found that the hypermethylation of CD24, CD133, and CD147 genes is associated with their downregulation in OSCC cell lines and primary tumors. Furthermore, the study found that the methylation levels of CD133 and CD147 are associated with poor prognosis in patients with OSCC. The limitation of the study was that a small number of OSCC cell lines and primary tumor tissues were included, thus further studies are needed to confirm the findings [22].

OLP belongs to the oral potentially malignant disorders and studies are necessary to establish the risk of malignant transformation. CSCs are considered to be the mediators of such a transition to cancer. Based on the aforementioned studies, our original research aims to illustrate, through immunohistochemistry, the pattern of expression of three CSCs' biomarkers (ALDH/SOX/CD147) in OLP compared to OSCC and possibly uncover, unknown so far, intracorrelations.

## Materials and methods

Preparation

To monitor the expression profile of ALDH1/2, SOX2 and CD147, an immunohistochemical detection took place in 24 samples of OLP (10 reticular OLPs (ROLPs) and 14 erosive OLPs (EOLPs)) and in 21 OSCC samples of all degrees of differentiation. To complete the above procedure, immunohistochemistry was used in a semiquantative manner. The paraffin-embedded tissue samples were selected from the archives of the Department of Oral Medicine/Pathology, School of Dentistry, Aristotle University of Thessaloniki, Greece, between 2014 and 2019, from biopsies performed in this department as well as from the Oral and Maxillofacial Surgery Clinic of G. Papanikolaou General Hospital, Aristotle University, and Oral and Maxillofacial Surgery Clinic of St Luke Hospital, Thessaloniki, Greece. 

Inclusion criteria

The inclusion criterion was the presence of an adequate quantity of paraffin-embedded tissue, whereas the exclusion criterion was the opposite. 

Sample size

The study was retrospective and based on the availability of archived tissue specimens that fulfilled the predefined inclusion criteria. Consequently, the sample size was determined by the number of eligible cases available during the study period rather than by a prospective recruitment target. A formal a priori sample size calculation was not performed because no previous studies using our specific methodology, markers, scoring system, and outcome measures were available to provide reliable estimates of effect size. Such estimates are required for meaningful power calculations. Under these circumstances, any power analysis would have been based on arbitrary assumptions and therefore would not have provided a robust justification for the sample size. The unequal group sizes reflect the natural distribution of eligible cases within the archival material and the differing availability of specimens meeting the inclusion criteria. No intentional allocation or matching was performed, as the study was observational and retrospective in design. All available eligible specimens were included to maximize statistical power and minimize selection bias.

OLP immunohistochemical research frequently includes between 10 and 30 cases per group, with larger cohorts (>50 OLP cases) being relatively uncommon. The literature therefore demonstrates that sample size is often constrained by the availability of suitable archived specimens rather than by prospective recruitment targets. Indicatively, we report the sample sizes of published immunohistochemical studies on OLP [23-26].

Ethical clearance

The study was conducted in accordance with the guidelines of the Research and Ethics Committee of Aristotle University, School of Dentistry and the Declaration of Helsinki II. The present study was approved by the Ethics Committee of the School of Dentistry, Aristotle University of Thessaloniki, Greece at its meeting on July 3, 2019, with the protocol number 8/03.07.2019. 

Process

The sections were mounted on slides (Polysine and Superfrost Plus, Thermo Scientific Menzel Gläser, Germany) for the application of the immunohistochemical method, and the corresponding hematoxylin sections were compared. Using the Dako EnVision FLEX+ system (Dako Denmark A/S, Denmark) as a secondary stain detection system and the chromogenic agent application (DAB EnVision, Dako Denmark A/S, Denmark), the anti-CD147 (sc-21746, Santa Cruz Biotechnology, USA), anti-ALDH1/2 (sc-166362, Santa Cruz Biotechnology, USA) and anti-SOX2 (sc-365823, Santa Cruz Biotechnology, USA) were used at a dilution of 1:100 when using the immunohistochemical technique to further process the sections. The findings of the immunohistochemical staining evaluation were then observed and documented by looking at the sections under a microscope. An Olympus CX31 microscope (Olympus LS, Japan) and an Olympus SC30 camera (Olympus Soft Imaging Solutions, Germany) were used for microscopy and data capture. 

Evaluation 

ALDH1/2, SOX2 and CD147 expressions were evaluated through a scale of 0 to 2 depending on the percentage of positive epithelial cells. 0 is equivalent to less than 5% positive cells. 1 is equivalent to the presence of positive cells in the lower third of the epithelium, which includes approximately the basal and suprabasal cell layers. 2 is equivalent to the presence of positive cells to any higher degree compared to 1. The evaluation was conducted by the researchers in collaboration with one another to avoid errors due to subjective biases. The observations were independently recorded by the investigators while maintaining the anonymity of all samples. Any discrepancies between the investigators were resolved through evaluation by our supervisor, A.P. Cells with stem cell-like features are expected to be found in the basal cell layer; therefore, the positive staining to that cell layer does not have necessarily clinical implications. Therefore, only a grade 2 staining could be translated into a finding with clinical significance. The staining is considered to be successful when the cytoplasm, nucleus or membrane is colored brown.

Scoring system justification

Compared to H-score and immunoreactive score (IRS), our scoring system seems simpler but it fits better to the aims. The biological and histopathological significance of staining in our study is determined primarily by its distribution within the epithelial thickness, rather than by the overall percentage of positive cells or staining intensity. H-score and IRS are therefore valuable semiquantitative methods, but they may not adequately capture the specific biological question addressed by our study.

Statistics

Statistical analysis was performed using Statistical Package for the Social Sciences (SPSS) software (2017) with a Pearson’s chi-square test or Fisher’s exact test depending on the sample size. The significance level was set at 0.05 (p=0.05).

## Results

With regard to ALDH1/2, the statistical analysis showed statistically significantly higher expression in OSCC than in EOLP (p=0.019) and in ROLP (p<0.001). With regard to SOX2 staining, the statistical analysis showed statistically significantly higher expression in EOLP than in ROLP (p=0.024), as well as in OSCC than in ROLP (p=0.012). With regard to CD147 staining, the statistical analysis showed statistically significantly higher expression in EOLP than in ROLP (p=0.019). The rest of the comparisons yielded no statistically significant results. Table 1 summarizes the grades of staining per sample, displaying the epidemiological characteristics of the patients from which each sample derived.

**Table 1:** Summary of the staining grades of the samples, as well as, the epidemiological characteristics of the patients, from which the samples derived ROLP: Reticular oral lichen planus; EOLP: Erosive oral lichen planus; OSCC: Oral squamous cell carcinoma

SOX 2	ALDH1/2	CD147	Disorder	Localization	Gender	Age
1	2	1	ROLP	Tongue	Male	57
1	1	1	ROLP	Tongue	Male	77
1	1	1	ROLP	Tongue	Female	21
1	1	1	ROLP	Tongue	Female	50
1	2	1	ROLP	Tongue	Female	57
1	1	1	ROLP	Cheek	Female	72
1	2	1	ROLP	Tongue	Female	38
1	1	1	ROLP	Cheek	Male	73
1	1	1	ROLP	Cheek	Female	49
1	1	1	ROLP	Cheek	Female	42
1	2	2	EOLP	Gingiva	Female	77
1	2	2	EOLP	Cheek	Female	77
1	2	2	EOLP	Tongue	Female	59
2	2	2	EOLP	Cheek	Female	54
2	2	1	EOLP	Palate	Female	55
2	2	0	EOLP	Cheek	Female	49
2	2	2	EOLP	Cheek	Female	72
2	1	2	EOLP	Cheek	Female	76
1	2	1	EOLP	Cheek	Female	58
1	1	1	EOLP	Cheek	Female	64
1	2	1	EOLP	Cheek	Male	56
2	2	2	EOLP	Cheek	Male	56
1	1	1	EOLP	Gingiva	Female	26
1	1	1	EOLP	Cheek	Female	37
2	2	2	OSCC	Tongue	Female	47
2	2	1	OSCC	Tongue	Female	53
1	2	1	OSCC	Cheek	Male	45
2	2	1	OSCC	Tongue	Female	77
1	2	1	OSCC	Cheek	Male	66
0	2	1	OSCC	Cheek	Male	61
0	2	1	OSCC	Mouth floor	Female	76
2	2	1	OSCC	Tongue	Male	75
1	2	1	OSCC	Tongue	Female	79
1	2	1	OSCC	Cheek	Female	78
2	2	1	OSCC	Tongue	Female	72
1	2	0	OSCC	Tongue	Male	52
0	2	0	OSCC	Tongue	Male	60
2	2	1	OSCC	Tongue	Female	77
0	2	0	OSCC	Tongue	Female	80
1	2	1	OSCC	Mouth floor	Female	82
2	2	1	OSCC	Lip	Male	58
2	2	2	OSCC	Lip	Male	77
2	2	1	OSCC	Tongue	Female	73
1	2	2	OSCC	Tongue	Female	67
2	2	1	OSCC	Tongue	Male	43

**Figure 1:**
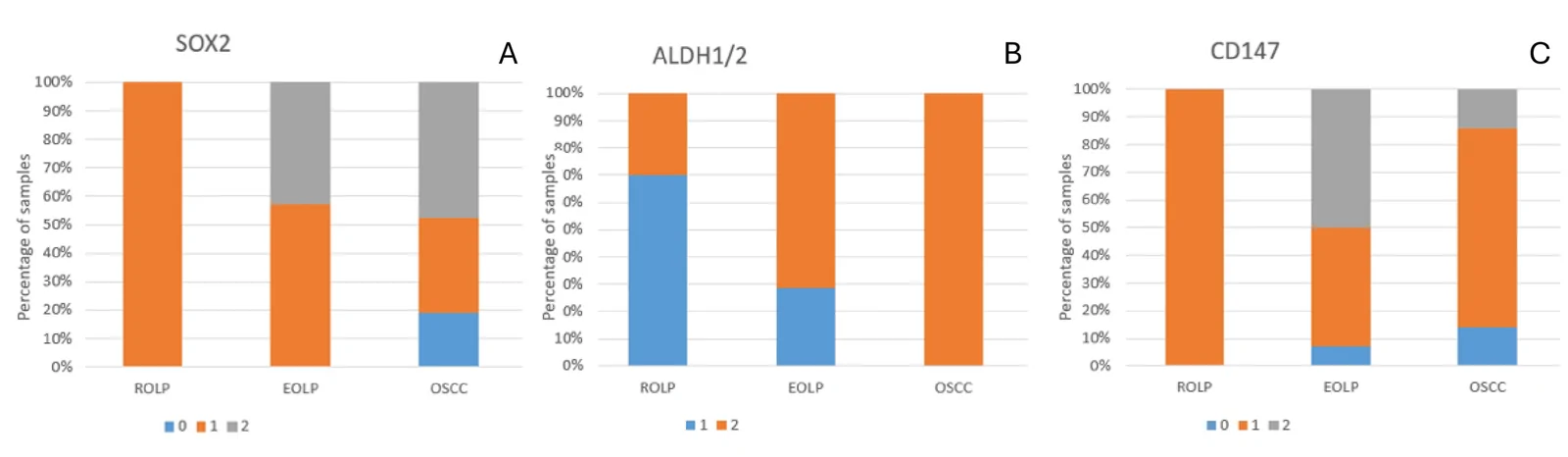
The percentagewise SOX2, ALDH1/2 and CD147 expression in ROLP, EOLP and OSCC A: Regarding SOX2 expression, the degree of staining (0-1-2) corresponds to different colors (blue-orange-grey), thus displaying the percentage of samples per disorder (ROLP, EOLP, OSCC) per degree of staining. B: Regarding ALDH1/2 expression, the degree of staining (1-2) corresponds to different colors (blue-orange), thus displaying the percentage of samples per disorder (ROLP, EOLP, OSCC) per degree of staining. C: Regarding CD147 expression, the degree of staining (0-1-2) corresponds to different colors (blue-orange-grey), thus displaying the percentage of samples per disorder (ROLP, EOLP, OSCC) per degree of staining. ROLP: Reticular oral lichen planus; EOLP: Erosive oral lichen planus; OSCC: Oral squamous cell carcinoma

Figure 1 summarizes the expression of each marker per disorder.

Tables 2-4 summarize the statistical analysis per marker.

**Table 2:** SOX2 expressions’ statistical analysis among ROLP, EOLP, and OSCC Fisher's exact test was implemented apart from the exceptions noted with * where Pearson chi-square was applied. ROLP: Reticular oral lichen planus; EOLP: Erosive oral lichen planus; OSCC: Oral squamous cell carcinoma

SOX 2	ROLP	EOLP	OSCC
ROLP	-	0.024	0.012
EOLP	-	-	0.782*
OSCC	-	-	-

**Table 3:** ALDH1/2 expressions’ statistical analysis among ROLP, EOLP, and OSCC Fisher's exact test was implemented apart from the exceptions noted with * where Pearson chi-square was applied. ROLP: Reticular oral lichen planus; EOLP: Erosive oral lichen planus; OSCC: Oral squamous cell carcinoma

ALDH1/2	ROLP	EOLP	OSCC
ROLP	-	0.095	<0.001
EOLP	-	-	0.019
OSCC	-	-	-

**Table 4:** CD147 expressions’ statistical analysis among ROLP, EOLP, and OSCC Fisher's exact test was implemented apart from the exceptions noted with * where Pearson chi-square was applied. ROLP: Reticular oral lichen planus; EOLP: Erosive oral lichen planus; OSCC: Oral squamous cell carcinoma

CD147	ROLP	EOLP	OSCC
ROLP	-	0.019	0.533
EOLP	-	-	0.053
OSCC	-	-	-

The following figures depict the pattern of expression of ALDH1/2, SOX2 and CD147 in OLP and OSCC cases (Figures 2-11).

**Figure 2:**
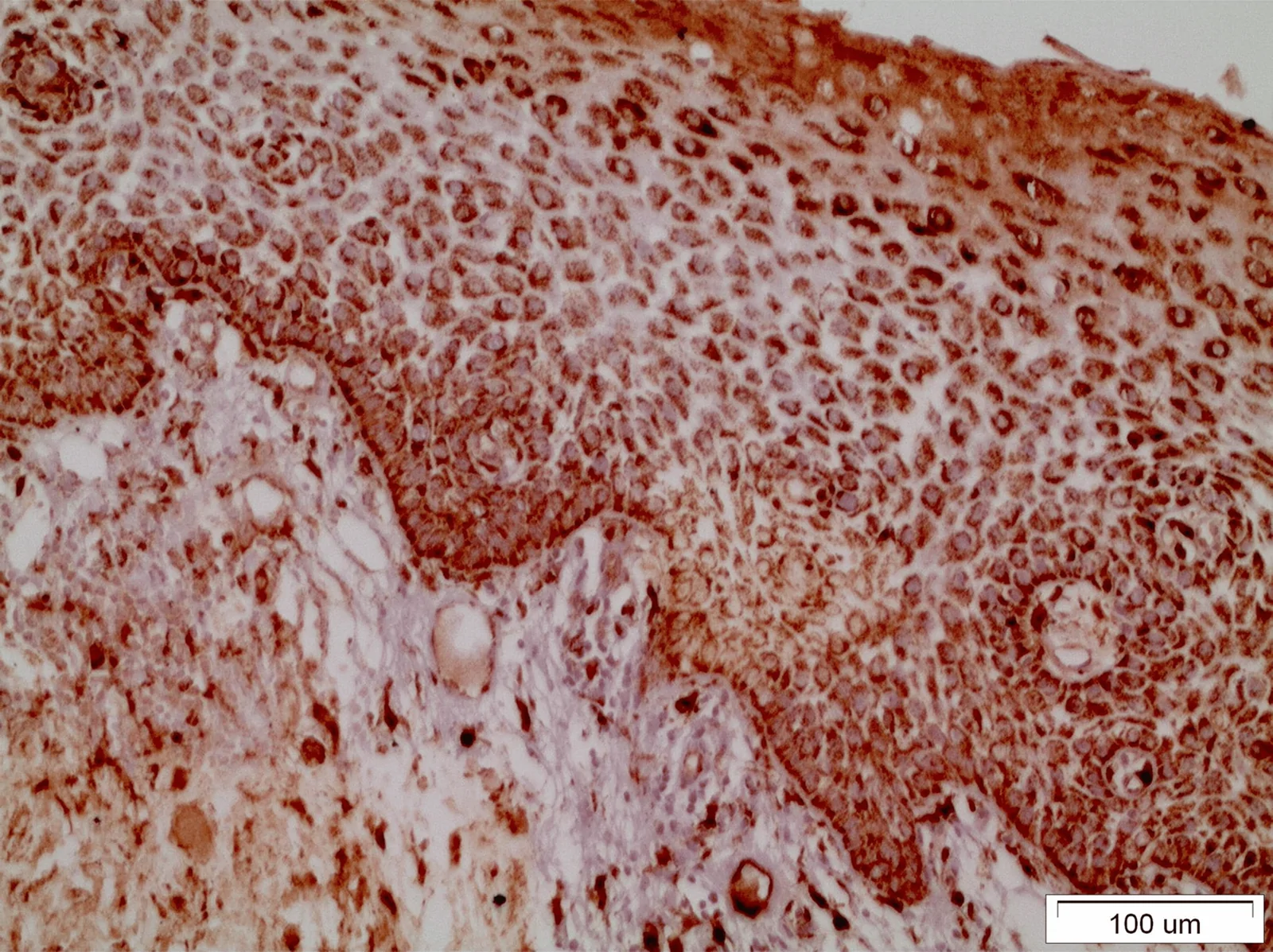
Histological depiction of the neighboring oral mucosa epithelium in an OSCC case where a strong ALDH positivity is noticed throughout the epithelium (X20) OSCC: Oral squamous cell carcinoma

**Figure 3:**
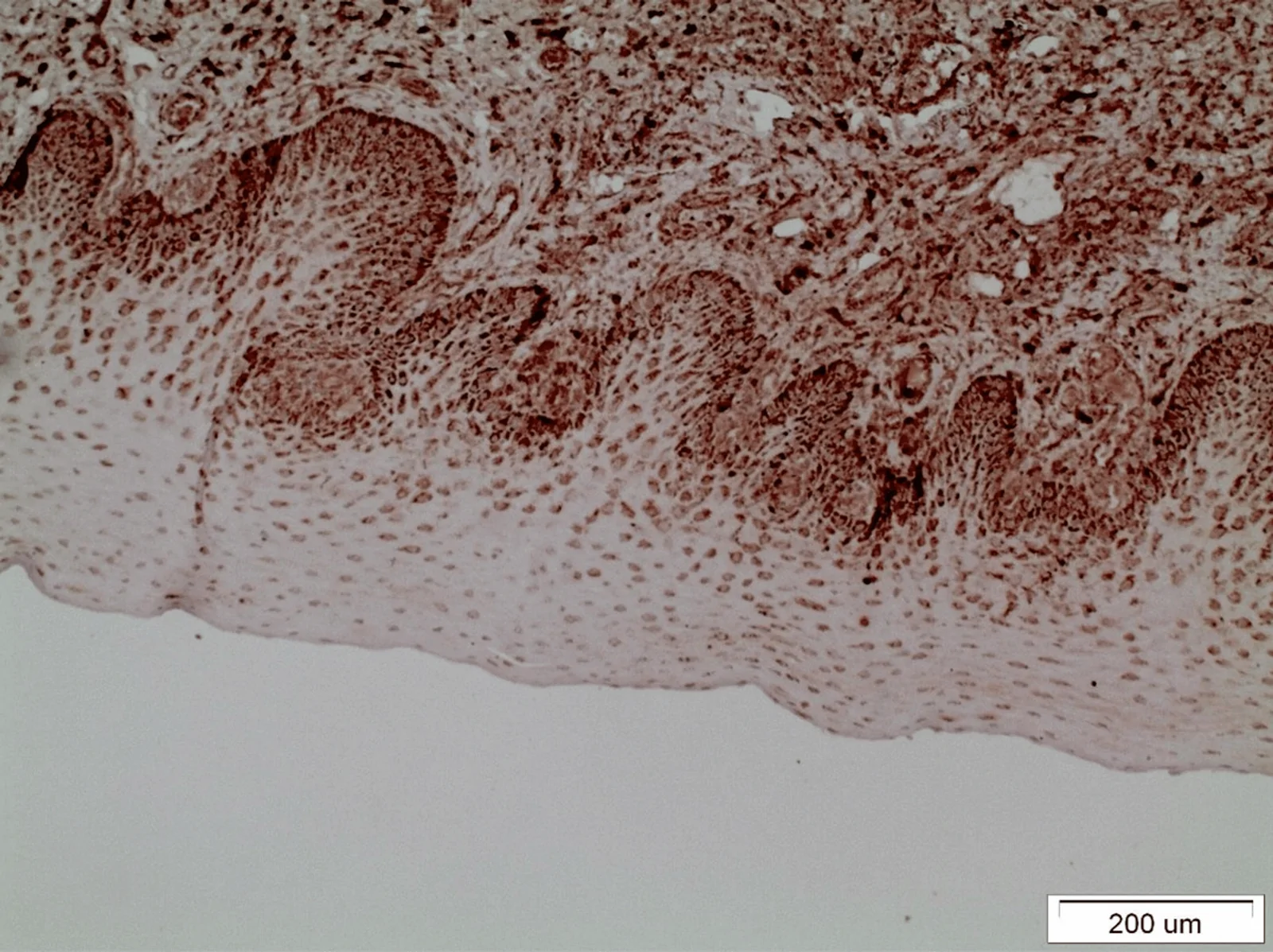
Histological depiction of an EOLP case where ALDH positive cells are noticed mostly in the basal cell layer, and then in the suprabasal cell layer (X10) EOLP: Erosive oral lichen planus

**Figure 4:**
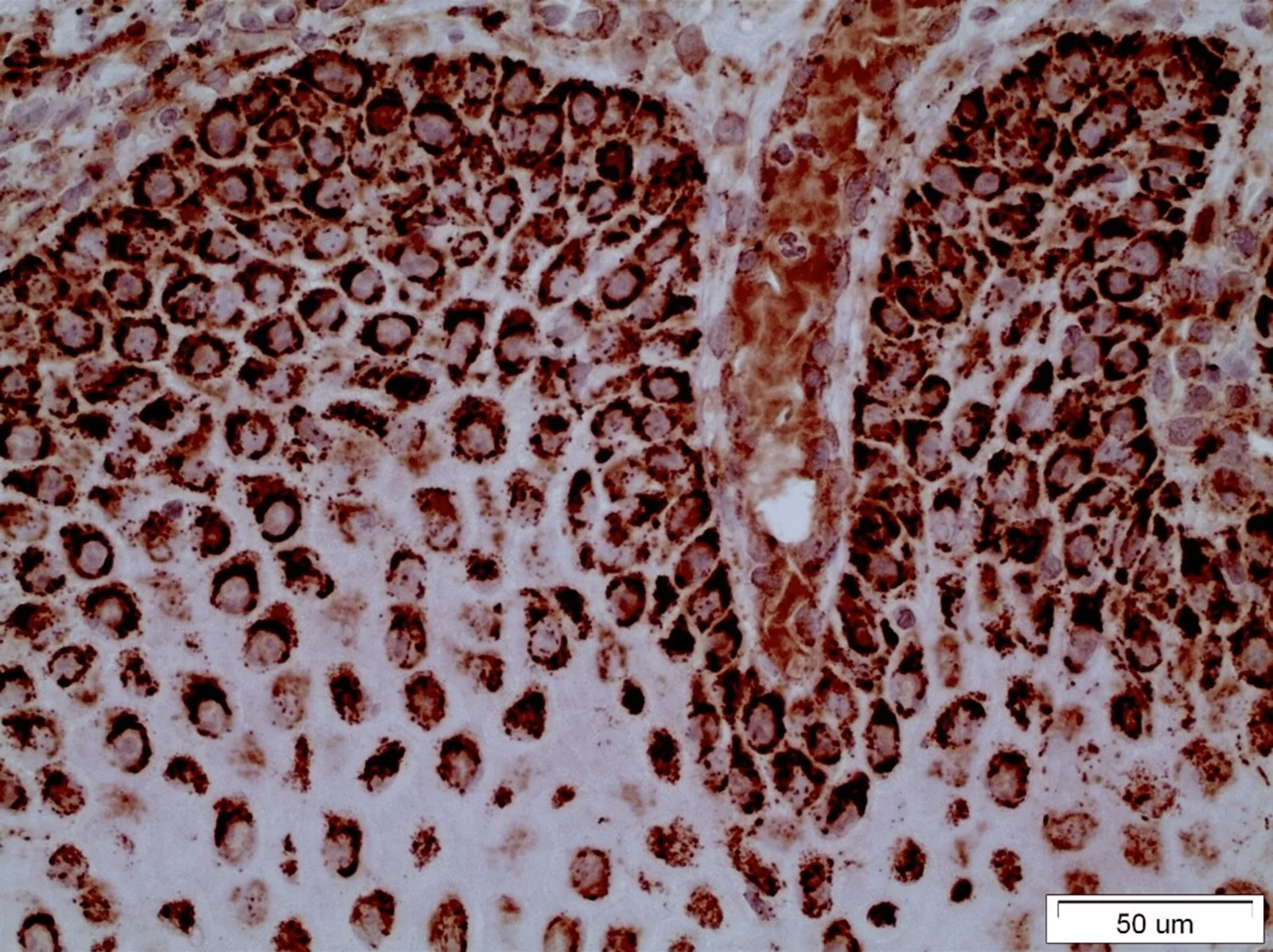
Histological depiction of an EOLP case where ALDH positive cells are noticed in the basal cell layer (X40) EOLP: Erosive oral lichen planus

**Figure 5:**
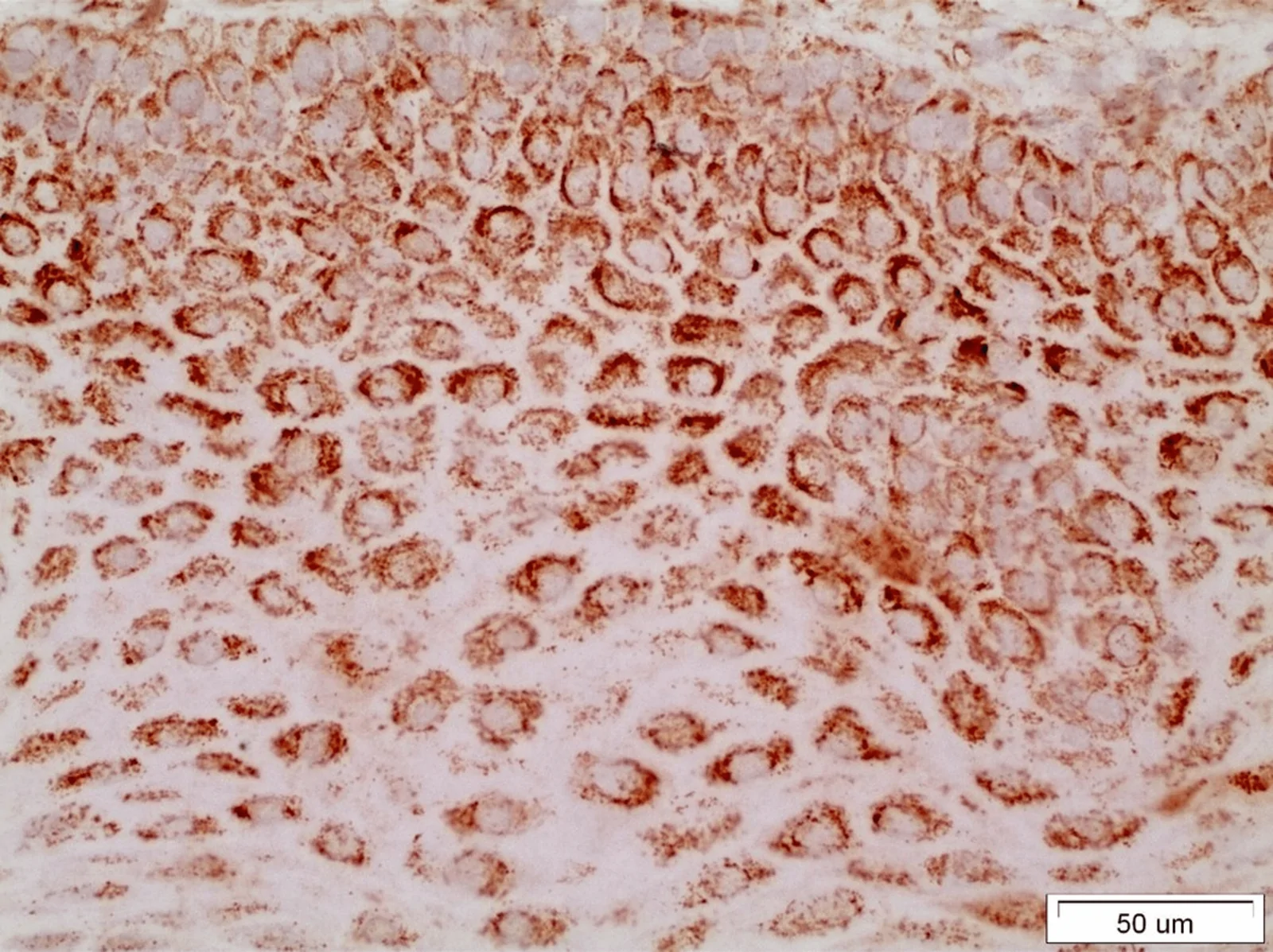
Histological depiction of a ROLP case where, relatively mildly stained, ALDH positive cells are noticed in the basal and suprabasal cell layers (X40) ROLP: Reticular oral lichen planus

**Figure 6:**
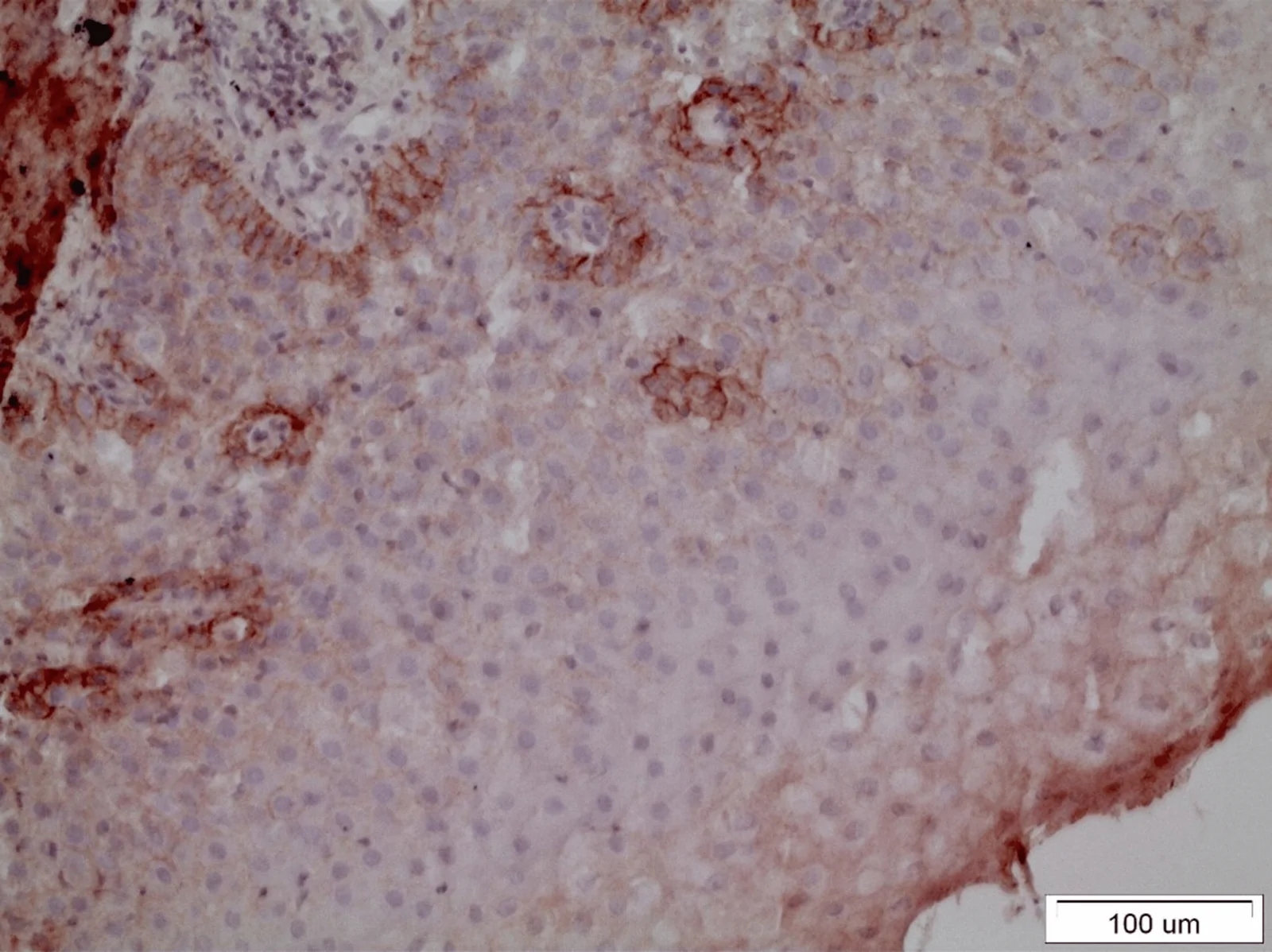
Histological depiction of the neighboring oral mucosa epithelium in an OSCC case where dispersed CD147 positive cells are noticed in the basal cell layer and very dispersely in the overlying cell layers (X20) OSCC: Oral squamous cell carcinoma

**Figure 7:**
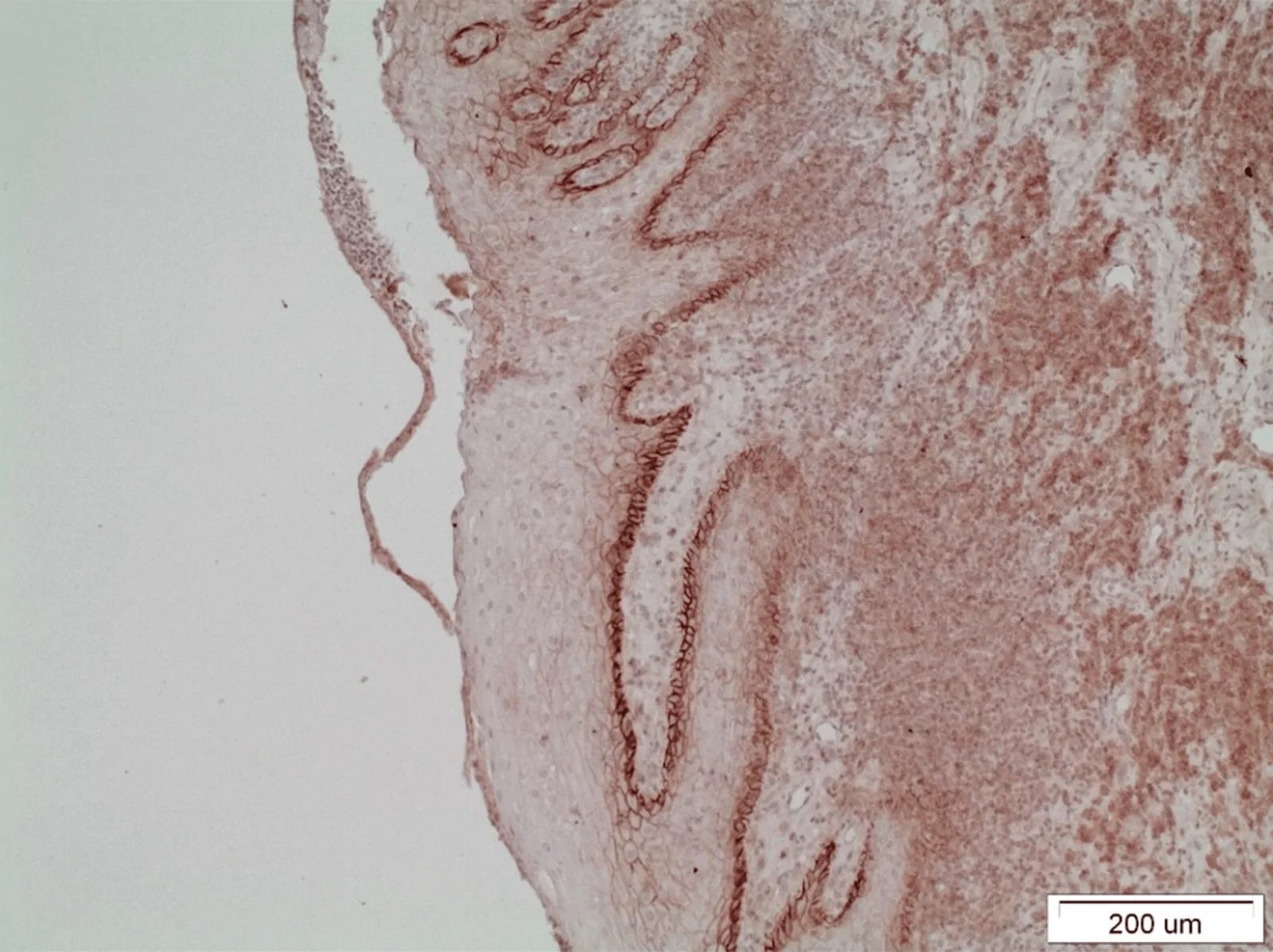
Histological depiction of an EOLP case where a very distinct CD147 positivity is noticed in the basal cell layer with few positive cells present in the other layers (X10) EOLP: Erosive oral lichen planus

**Figure 8:**
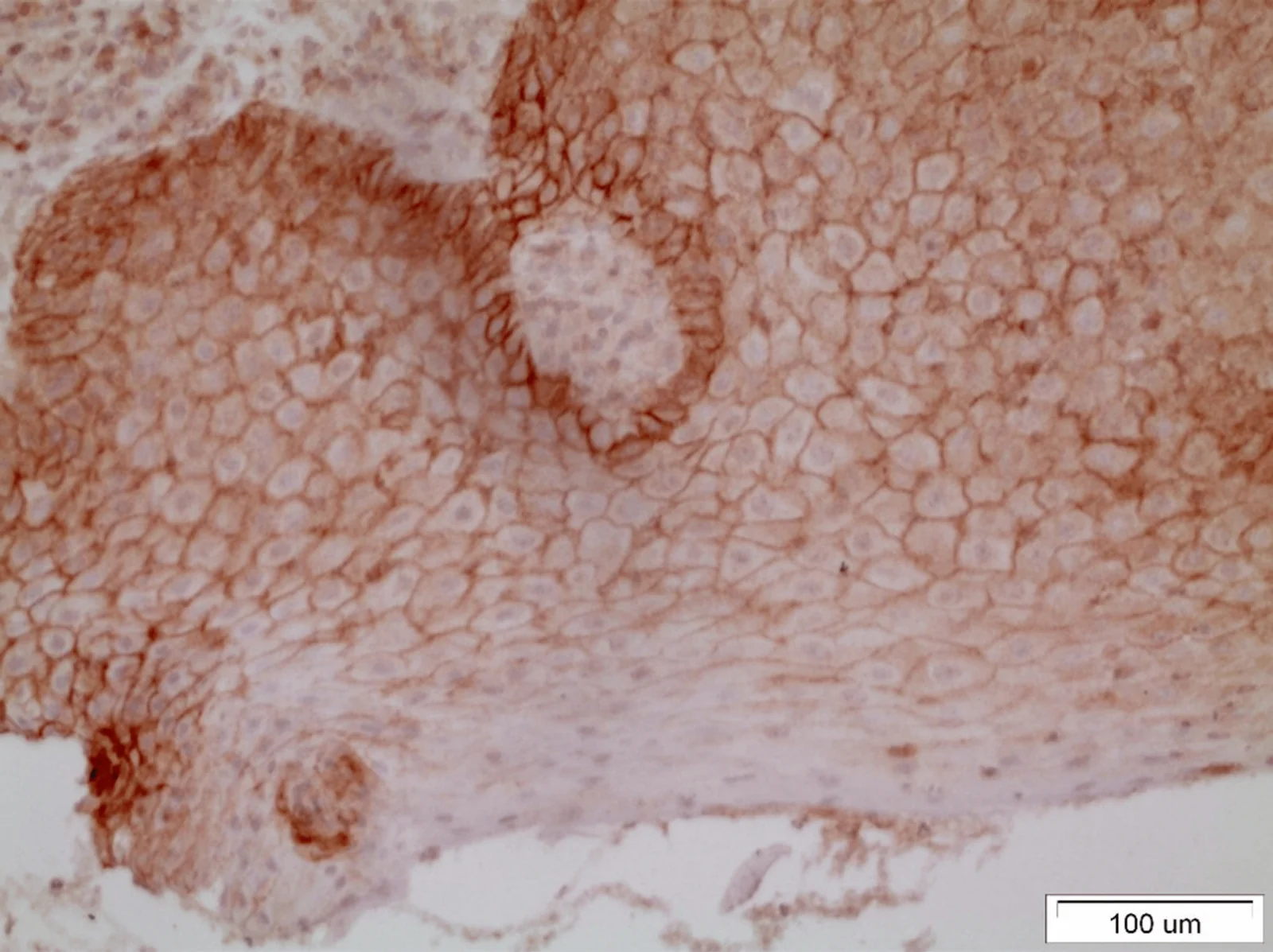
Histological depiction of a ROLP case where a relatively milder CD147 positivity is noticed in the lower one-third of the epithelium ROLP: Reticular oral lichen planus

**Figure 9:**
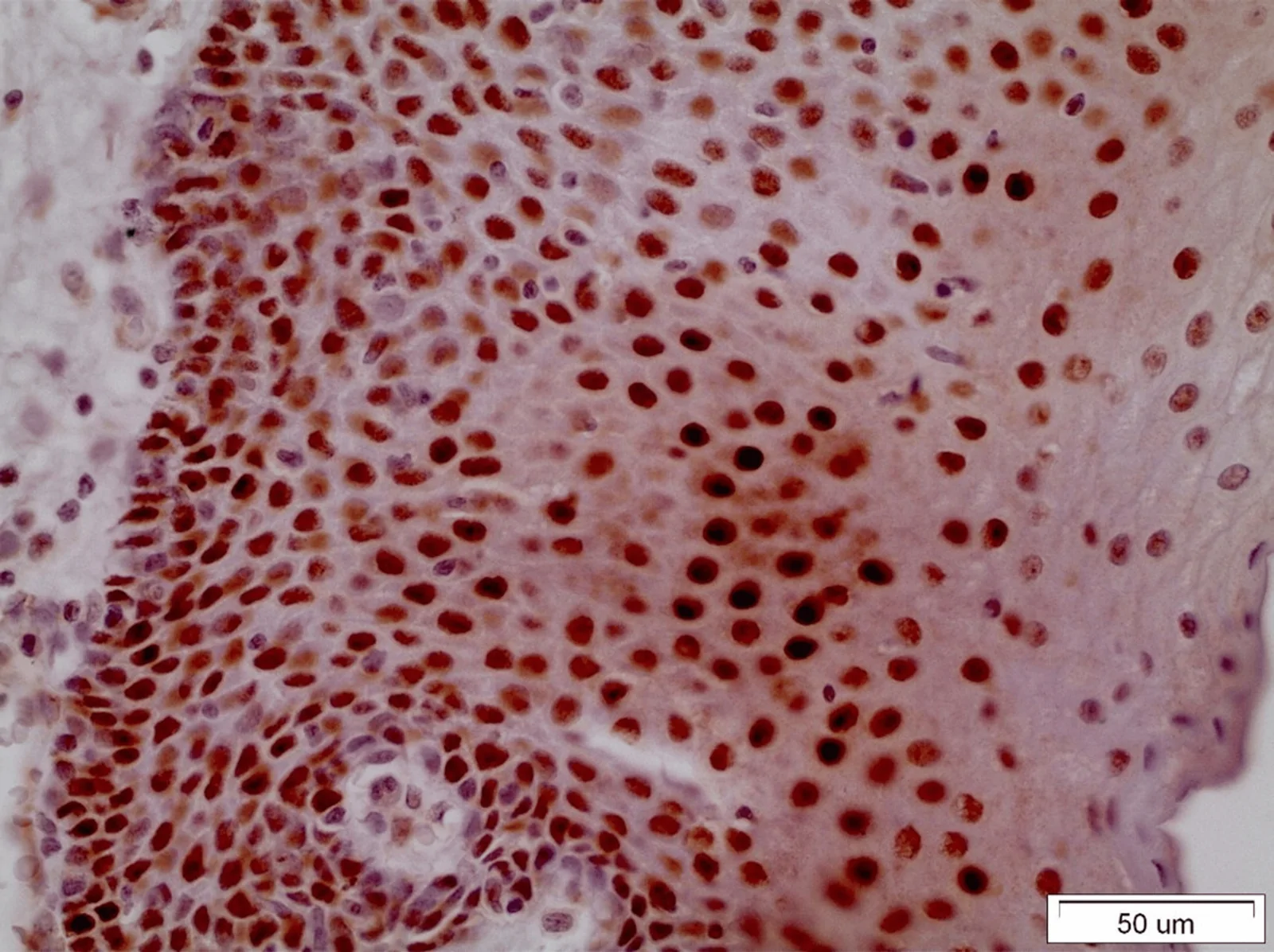
Histological depiction of the neighboring oral mucosa epithelium in an OSCC case where strong SOX positivity is noticed in more than two-thirds of the epithelium OSCC: Oral squamous cell carcinoma

**Figure 10:**
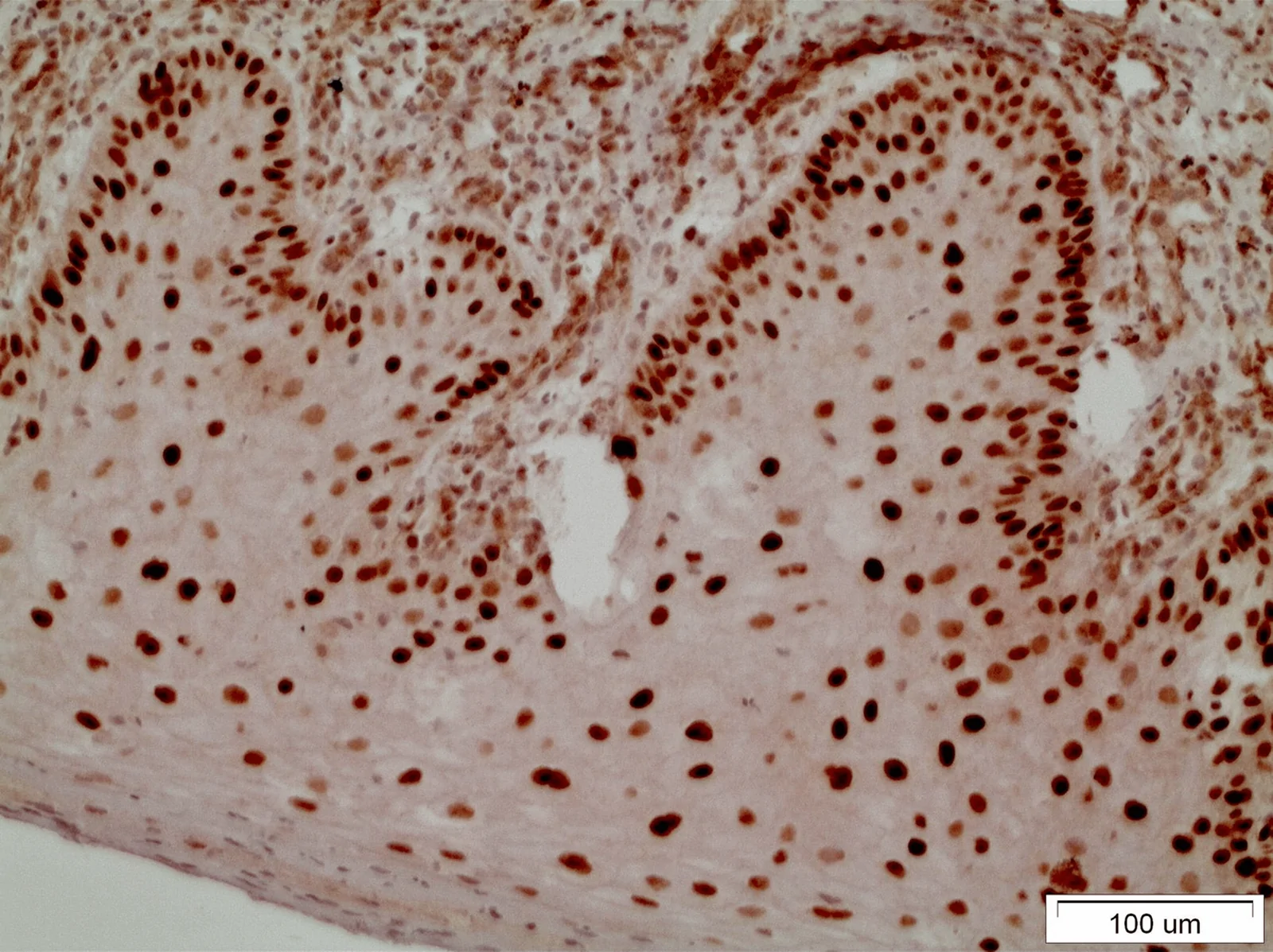
Histological depiction of an EOLP case where an overall strong SOX positivity is noticed. The majority of positive cells is noticed in the basal cell layer. EOLP: Erosive oral lichen planus

**Figure 11:**
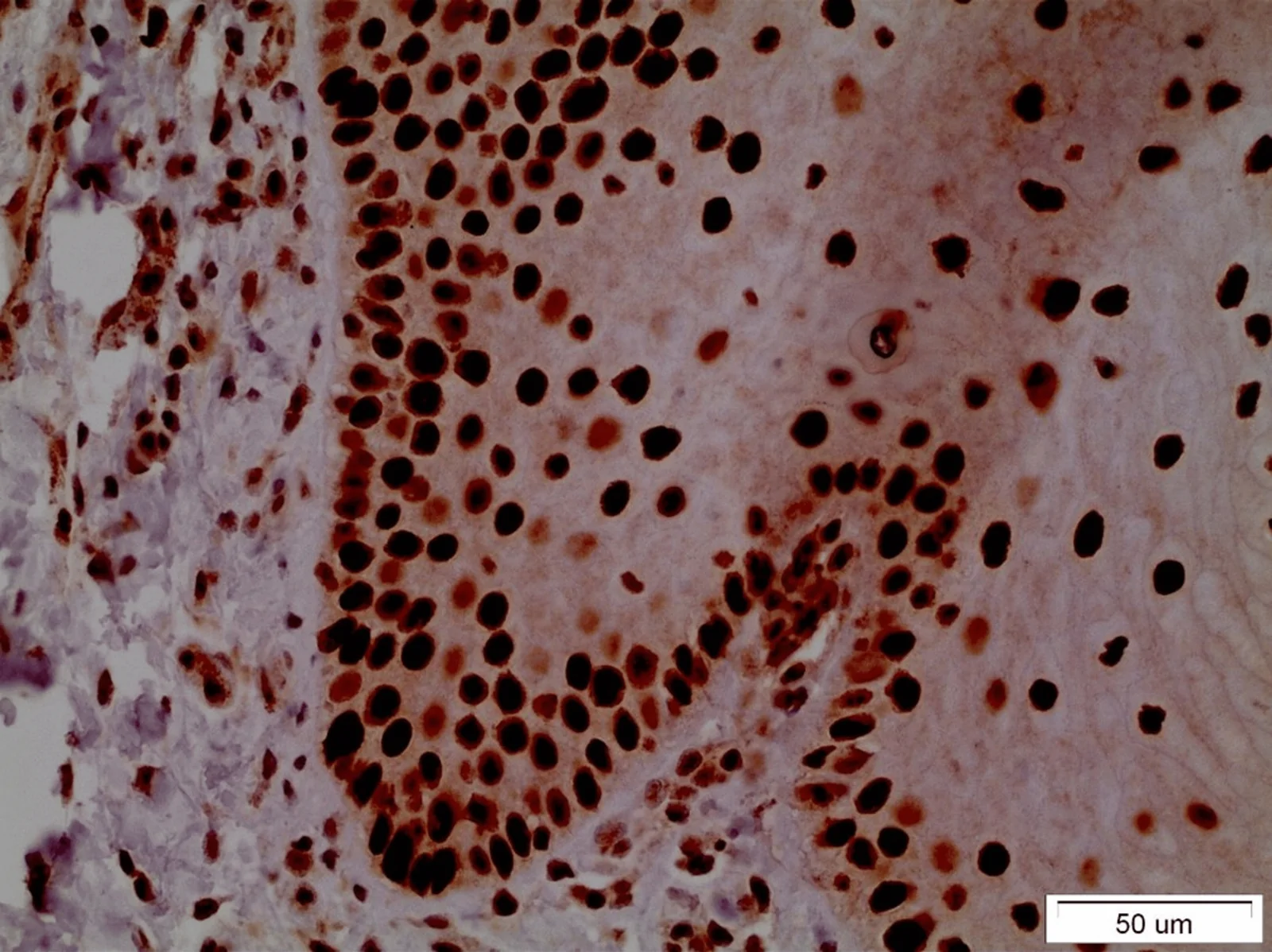
Histological depiction of a ROLP case where strong SOX positivity is noticed exclusively in the basal and parabasal cell layer ROLP: Reticular oral lichen planus

Therefore, we reach the following conclusions: ALDH1/2 acts as a strong malignancy marker (OSCC>EOLP, ROLP). SOX2 acts as an early transformation marker (EOLP & OSCC> ROLP). CD147 acts as an early inflammatory marker (EOLP>ROLP). The pattern of ALDH1/2 appeared to be both cytoplasmic and membranous, of CD147 membranous and of SOX2 nuclear as well as perinuclear. The basal cell layer was positive which is expected due to its stem cell like features and the inflammation present in both OLP and OSCC. The SOX2 expression in upper layers in EOLP (Figure 10) may be interpreted as the presence of oral epithelial stem cells where they are not supposed to be. Possibly, the inflammatory substrate of OLP creates an overall dysregulation thus providing the background for malignant transition. The results on ALDH1/2 mean that this marker may not be applied to successfully differentiate the relative risk of malignant transition among lichenoid lesions. CD147 seems to as a more indirect marker. EOLP outperformed ROLP meaning that inflammation in EOLP is statistically significantly higher and chronic inflammation continually releases chemicals like cytokines that prompt cells to divide and repair. Over time, this environment leads to DNA damage and mutations.

## Discussion

These findings may be interpreted in a multitude of ways. The characteristic higher expression of CD147 in EOLP compared to ROLP, suggests the possible presence of neoplastic cells with certain CSC characteristics in erosive lichenoid lesions. This supports the theory of a more direct association between OLP and OSCC, where the presence of EOLP is interpreted as the proof of presence of specific CSCs (due to high cellular stress) [27]. On the other hand, we know from the literature that OLP involves inflammatory infiltrate [28], and chronic inflammation is a known co-founding or co-occurring factor for cancer [29,30]. CD147 is up regulated in inflammation therefore its presence may not prove the presence of cancer but just indicate the presence of inflammation which in turn may lead in the long term to cancer [31]. These scenarios are not mutually exclusive; possibly both are relevant depending on other genetic or environmental factors. 

The same theories support the characteristic higher expression of SOX2 in EOLP and OSCC compared to ROLP. Again, neoplastic cells with certain CSC characteristics may be present in erosive lichenoid lesions. SOX constitutes an embryonic stem cell marker and is expected to be expressed by cells with stem cell like features [32]; the basal and suprabasal cell layers contain stem cell like cells [33] and may be positive under normal circumstances. However, in grade 2 staining, the presence of aberrant cells which exhibit stem cell like characteristics in other cell layers, is evidence based [34]. ALDH1/2 expression is in accordance with the literature since its expression is expected to be higher in cancerous lesions than in lichenoid [35].

In any case, the clinical use of CSCs' biomarkers as prognostic factors requires further experimental evaluation in larger number of samples [36]. Compared to previous immunohistochemical experiments from our lab [37-42], we altered a few parameters. Our earlier grading had applied a scoring range 0-3, by dividing the epithelium into thirds. It made sense on histological level; however, in practice, the basal cell layer has much more stem cell-like cells than the rest of the layers. Therefore, this first third, when positive, has fewer biological repercussions than the others. Thereby, we modified the scoring range from 0-3 to 0-2, and a score of 2 includes any positive staining above the lower third. Furthermore, this time, we grouped all OSCC samples together, while dividing OLP in different subtypes, since in previous experiments, the degree of differentiation of OSCC did not seem to play a key role whereas the OLP subtype did [37-42]. Our observations so far suggest that there may be different immunohistochemical profiles in OSCC samples, possibly differentiating the cancerous locus from the adjacent, normal appearing, oral mucosa epithelium which surrounds it. These observations could be explained by the field cancerization theory which suggests that the tissue develops genetic/epigenetic alterations, which create a field of cells, histologically either normal or dysplastic, which are already molecularly altered [43]. Therefore, by definition, even the normal appearing surrounding mucosa expresses biomarkers that may precede the emergence of cancer. OLP patients who develop dysplasia and OSCC are at risk of numerous, multifocal neoplastic events due to the field cancerization [44].

Future prospects

Furthermore, CSC’s biomarkers could be utilized to classify OLP cases, by establishing a higher probability of malignant transition when positive. The absence of definitive clinical criteria distinguishing prognostically insidious cases from less insidious ones could be covered by the implementation of immunohistochemical criteria. Non-invasive techniques like salivary biomarker testing and liquid biopsy, while still insufficient, may be proven fruitful in conjunction with immunohistochemistry regarding the better classification of potentially malignant lesions.

Limitations

The limitations of this study include the single institutional design, the small sample size, with the unevenly sized groups and the relatively simple - compared to H-score and IRS - scoring system (because it was deemed more appropriate for the specific aims of this study).

## Conclusions

OLP, one of the most common oral potentially malignant disorders, requires prompt diagnosis and a regular follow-ups protocol. The possibility of malignant transformation is relatively small; however, its exact estimation demands further investigation. The distinctively increased expression of CD147 in EOLP as opposed to ROLP raises the possibility that erosive lichenoid lesions contain neoplastic cells with specific CSC traits. The distinctively increased expression of SOX2 in OSCC and EOLP as opposed to ROLP raises the possibility that erosive lichenoid lesions contain malignant cells with specific CSC traits. In any event, additional experimental testing in a greater number of samples is necessary for the clinical application of CSC biomarkers as prognostic variables. The evidence so far supports the progressive implementation of ALDH, SOX and CD147 in clinical practice. New biomarkers may shed some light on the underlying mechanisms that mediate the transition from oral potentially malignant disorders to OSCC.
